# Is fMRI “noise” really noise? Resting state nuisance regressors remove variance with network structure

**DOI:** 10.1016/j.neuroimage.2015.03.070

**Published:** 2015-07-01

**Authors:** Molly G. Bright, Kevin Murphy

**Affiliations:** a Division of Clinical Neurology, School of Medicine, University of Nottingham, Nottingham, United Kingdom; b Sir Peter Mansfield Imaging Centre, School of Physics, University of Nottingham, Nottingham, United Kingdom; c Cardiff University Brain Research Imaging Centre (CUBRIC), School of Psychology, Cardiff University, Cardiff, United Kingdom

**Keywords:** FMRI, Resting state, Connectivity, Noise correction, Motion, Regression

## Abstract

Noise correction is a critical step towards accurate mapping of resting state BOLD fMRI connectivity. Noise sources related to head motion or physiology are typically modelled by nuisance regressors, and a generalised linear model is applied to regress out the associated signal variance. In this study, we use independent component analysis (ICA) to characterise the data variance typically discarded in this pre-processing stage in a cohort of 12 healthy volunteers. The signal variance removed by 24, 12, 6, or only 3 head motion parameters demonstrated network structure typically associated with functional connectivity, and certain networks were discernable in the variance extracted by as few as 2 physiologic regressors. Simulated nuisance regressors, unrelated to the true data noise, also removed variance with network structure, indicating that any group of regressors that randomly sample variance may remove highly structured “signal” as well as “noise.” Furthermore, to support this we demonstrate that random sampling of the original data variance continues to exhibit robust network structure, even when as few as 10% of the original volumes are considered. Finally, we examine the diminishing returns of increasing the number of nuisance regressors used in pre-processing, showing that excessive use of motion regressors may do little better than chance in removing variance within a functional network. It remains an open challenge to understand the balance between the benefits and confounds of noise correction using nuisance regressors.

## Highlights

• Data variance removed by nuisance regressors contains network structure.
• Simulated regressors unrelated to noise also extract data with network structure.
• Random sampling of original data (as few as 10% of volumes) reveals robust networks.
• After optimal number, motion regressors remove similar variance as simulated ones.
• Excessive nuisance regressors extract random signal variance with network structure.

## Introduction

Blood oxygenation-level dependent functional magnetic resonance imaging (BOLD fMRI) data can be analysed using a generalised linear model (GLM) to identify brain regions exhibiting signal changes time-locked to known stimuli. In addition to the activations of interest, BOLD data contain numerous noise signals reflecting thermal noise, scanner drift, magnetic field inhomogeneities, head motion, and human physiology. Some noise sources can be well modelled (e.g., using the results of rigid body registration algorithms or measuring respiration) and these nuisance regressors can be added to the GLM to improve sensitivity, specificity, and validity of activation maps (Bullmore et al., 1999; Lund et al., 2006).

Noise correction becomes increasingly important when mapping resting state signal fluctuations, which cannot be modelled by a known stimulus. Nuisance regressors derived from external measurements or from within the resting state dataset itself are removed prior to connectivity analysis (Murphy et al., 2013). Initial attempts at this noise correction approach have proved to be insufficient: numerous studies have demonstrated that head motion remains a critical confound in mapping functional connectivity despite removal of up to 12 motion-related nuisance regressors (Power et al., 2012; Satterthwaite et al., 2012; Van Dijk et al., 2012).

Recent evidence supports the use of up to 24 motion-related nuisance regressors in order to best remove this confound (Satterthwaite et al., 2013), while physiological noise is commonly removed using a combination of 12 or more cardiac and respiratory regressors (RETROICOR (Glover et al., 2000), blood gas levels (Bright and Murphy, 2013a; Wise et al., 2004), and respiratory and cardiac rates (Birn et al., 2008; Chang et al., 2009; Shmueli et al., 2007)).

This expanding number of nuisance regressors raises concerns. Measures of resting-state correlations do not typically account for the reduction in degrees of freedom associated with these noise correction approaches (Satterthwaite et al., 2013). Increasing the number of nuisance regressors also increases the risk of removing true signal of interest.

In this paper, we use independent component analysis (ICA) to characterise the portion of resting-state data typically removed and discarded during noise correction. First, we demonstrate that the signal variance removed by the regression of 24 head motion parameters can be decomposed into networks typically associated with functional connectivity. Several of these network structures can be observed in the variance extracted by as few as 3 head motion regressors or 2 physiologic regressors. Next, we consider simulated nuisance regressors, unrelated to the fMRI data: these simulated regressors also remove data with network structure, suggesting that any regressors may remove highly structured “signal” as well as “noise.” To directly address this, we show that sampling a small percentage of volumes at random from the original resting state data continues to produce robust network maps using ICA. Finally, we compare the variance explained by different numbers and combinations of true and simulated nuisance regressors within functional networks. The implications of these observations on our analysis and understanding of resting state fMRI are discussed.

## Methods

### Data acquisition

Twelve healthy subjects (aged 32 ± 6 years, 5 female) were scanned using a 3T GE HDx scanner (Milwaukee, WI, USA) equipped with an 8-channel receive head coil. An eyes-open resting state scan lasting 5.5 min was acquired using a BOLD-weighted gradient-echo echo-planar imaging sequence (TR/TE = 2000/35 ms; FOV = 22.4 cm; 35 slices, slice thickness = 4 mm; resolution = 3.5 × 3.5 × 4.0 mm^3^, 165 volume acquisitions). These data were collected as part of a larger study (Bright and Murphy, 2013b). A whole-brain high-resolution T1-weighted structural image was acquired (resolution = 1.0 × 1.0 × 1.0 mm^3^), for the purpose of image registration. Cardiac pulsations were recorded using the scanner finger plethysmograph. Expired gas content was continuously monitored via a nasal cannula, and O_2_ and CO_2_ data were recorded (AEI Technologies, PA, USA). This study was approved by the Cardiff University School of Psychology Ethics Committee, and all volunteers gave written informed consent.

### Data pre-processing

The resting-state functional data were volume registered, motion corrected, time-shifted to a common temporal origin, and brain extracted (AFNI, http://afni.nimh.nih.gov/afni (Cox, 1996)). The first five volumes, during which steady-state magnetisation was not yet achieved, were removed from the data. End-tidal CO_2_ and heart rate values were extracted from the physiological data (MATLAB, MathWorks, Natick, MA, USA) and convolved with an HRF and CRF (Chang et al., 2009), respectively. Derivatives of the 6 head motion regressors determined during motion correction were calculated, and the quadratic terms of these 12 regressors were derived.

The mean functional volume for each subject was registered to the corresponding high-resolution T1-weighted image, which was then normalised to the MNI-152 brain template (MNI152, nonlinearly derived, McConnell Brain Imaging Centre, Montreal Neurological Institute, McGill University, Montreal, Quebec, Canada). The combined transformation matrices were saved for later use.

### Construction of “noise” datasets

**Fig. 1:**
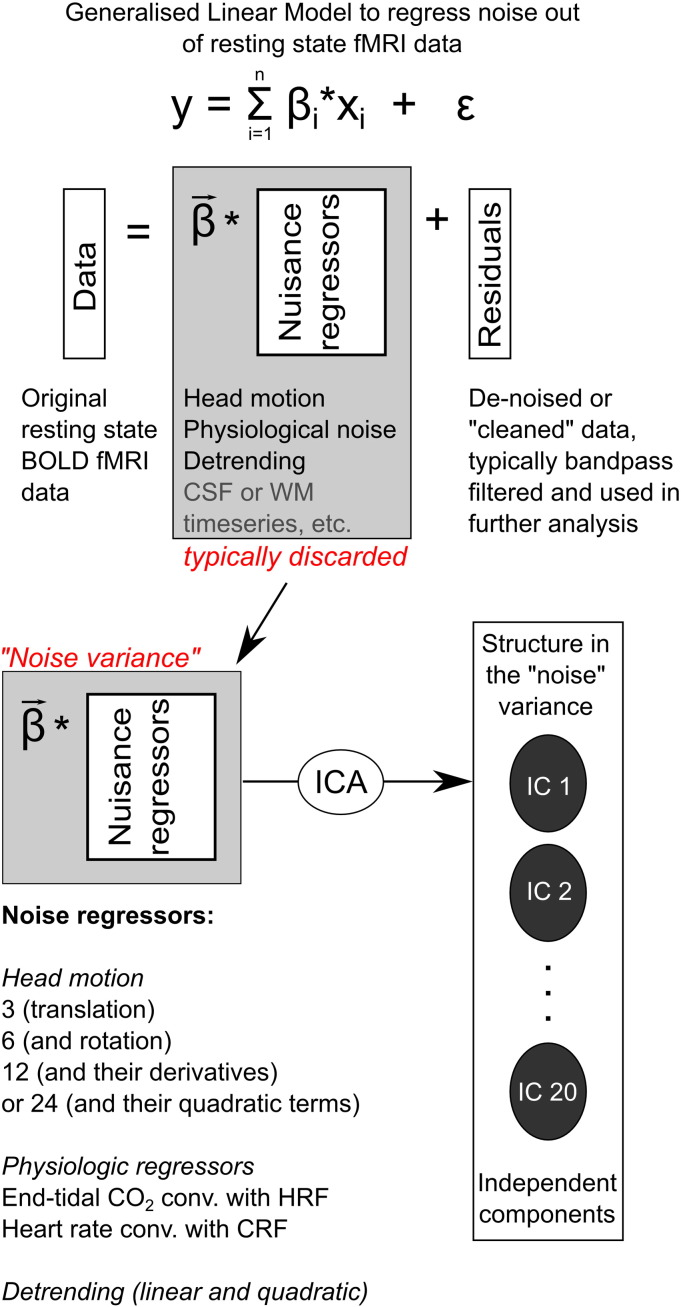
Schematic representing the construction of noise datasets and independent component analysis. Resting state BOLD fMRI data are input to a generalised linear model (GLM) where noise confounds are modelled by nuisance regressors. Typically, the residuals from this fitting procedure are considered to be “de-noised” and used for further connectivity analysis. Here we study the fit of the data to the nuisance regressors, and decompose this “noise dataset” using independent component analysis (ICA). We examined 3, 6, 12, or 24 head motion regressors (translations, rotations, their derivatives, and quadratic terms) or 2 physiologic regressors (end-tidal CO_2_ and cardiac rate), in addition to linear and quadratic detrending for signal drift removal, and fixed the dimensionality of the ICA output to be 20.

Three types of “noise” datasets were created using the original BOLD fMRI data and processed as shown schematically in Fig. 1.

#### Variance removed by true noise regressors

The head motion and physiologic noise for each resting state scan were used as regressors in a general linear model (GLM) using AFNI. The 4-dimensional dataset comprising the fit of the original data to these nuisance regressors was extracted, spatially smoothed (FWHM = 5 mm), and registered to MNI space using the transformations obtained earlier. This process was performed for motion-related noise, using 3 (x-, y-, z-translations), 6 (and three rotations), 12 (and their derivatives), or 24 (and their quadratic terms) head motion regressors (denoted Motion3, Motion6, Motion12, and Motion24 respectively), and for 2 physiologic regressors (end-tidal CO_2_ and heart rate). The use of 24 (or more) head motion regressors has been implemented in different ways throughout the literature (Friston et al., 1996; Satterthwaite et al., 2013; Yan et al., 2013a); in this paper, we adopt the method of Satterthwaite and colleagues (Satterthwaite et al., 2013). In all cases, linear and quadratic trends were included in the model to optimise fitting. The resulting datasets, which reflect variance typically removed during fMRI pre-processing steps (including linear and quadratic trends), are referred to as the “true noise” data. Physiologic regressors were always considered independently from the motion regressors in order to assess these distinct noise sources in isolation. For example, in the 3 regressor case, the true noise data reflects the 3 translation motion regressors and the linear/quadratic trends, but not the CO_2_ or heart rate variance.

#### Variance removed by simulated noise regressors

The true noise regressors were phase-randomised to create simulated noise regressors with similar frequency distributions to the true noise regressors, but with no relation to the measured head motion or physiology. To achieve this, the frequency spectra of the true noise regressors were obtained using a Fourier transform, and the phase of each frequency in half of the spectrum was randomised and mirrored before performing the inverse Fourier transform (this phase randomization was repeated until the temporal correlation with the true regressor was r < 0.1). The entire set of resulting time-series was then orthogonalised to the complete set of original regressors to make them independent from the true noise. These simulated regressors were input into a GLM in the same manner as the true noise regressors, and the resulting fits were also spatially smoothed (FWHM = 5 mm) and registered to MNI space and are referred to as “simulated noise” data. This process was repeated 10 times to allow us to test whether the true noise regressors behaved differently relative to simulated and unrelated ones. Examples of true and phase-randomised simulated noise regressors for one subject are provided in Supplementary Fig. S1.

The true noise regressors were phase-randomised to create simulated noise regressors with similar frequency distributions to the true noise regressors, but with no relation to the measured head motion or physiology. To achieve this, the frequency spectra of the true noise regressors were obtained using a Fourier transform, and the phase of each frequency in half of the spectrum was randomised and mirrored before performing the inverse Fourier transform (this phase randomization was repeated until the temporal correlation with the true regressor was r < 0.1). The entire set of resulting time-series was then orthogonalised to the complete set of original regressors to make them independent from the true noise. These simulated regressors were input into a GLM in the same manner as the true noise regressors, and the resulting fits were also spatially smoothed (FWHM = 5 mm) and registered to MNI space and are referred to as “simulated noise” data. This process was repeated 10 times to allow us to test whether the true noise regressors behaved differently relative to simulated and unrelated ones. Examples of true and phase-randomised simulated noise regressors for one subject are provided in Supplementary Fig. S1.

As an additional model of unrelated nuisance regressors, we also considered the noise datasets created by using the true noise regressors from another subject's data. Specifically, we used the noise regressors from Subject “N + 1” to remove variance from the fMRI data of Subject “N” (and using the regressors of Subject 1 for Subject 12). Correlations across true regressors, simulated regressors, and true regressors from an incorrect subject are presented, averaged across the cohort, in Supplementary Fig. S2.

As an additional model of unrelated nuisance regressors, we also considered the noise datasets created by using the true noise regressors from another subject's data. Specifically, we used the noise regressors from Subject “N + 1” to remove variance from the fMRI data of Subject “N” (and using the regressors of Subject 1 for Subject 12). Correlations across true regressors, simulated regressors, and true regressors from an incorrect subject are presented, averaged across the cohort, in Supplementary Fig. S2.

#### Variance achieved by randomly sampling the original data

Finally, we created reduced versions of the original data, retaining only a fraction of the signal variance. This provides a toy example imitating how a given set of nuisance regressors may randomly sample variance from the original fMRI data. A given percentage of the original data volumes (unfiltered, spatially smoothed and aligned to MNI space as described above) were randomly selected and replaced with the mean volume; this procedure retained 10, 20, 30, 40, 50, or 60% of the volumes, and was iterated 100 times. These datasets are referred to as “reduced” data (reduced10 through reduced60 as appropriate). We also considered reduced data consisting of the initial consecutive volumes only; these are referred to as “shortened” data (short10 through short60 as appropriate).

### Construction of “cleaned” datasets

The residuals from the fitting of the nuisance regressors to the resting state data, as indicated in Fig. 1, represent the de-noised or “cleaned” data. We constructed the cleaned datasets from the residuals of the fit to 24 true motion regressors. This dataset was then bandpass filtered (AFNI 3dBandpass, FFT-based filter, 0.02–0.1 Hz) to reflect current practises in the field of resting state pre-processing (Di Martino et al., 2013; Power et al., 2012, 2014), spatially smoothed, and registered to MNI space as described above. We also considered the unfiltered cleaned data, to test the influence of this processing step on network similarity.

### Network analysis

The cleaned data, true noise data, simulated noise data, reduced, and shortened data were analysed using spatial ICA as implemented in MELODIC as part of FSL (Beckmann et al., 2005; Jenkinson et al., 2012). The datasets of the 12 subjects were temporally concatenated, and dimensionality was fixed to output 20 components (except in the case of the short10 dataset, which had too few degrees of freedom to be decomposed into 20 components, where dimensionality was fixed to 10). The resulting networks were then compared as follows.

In the first instance identifying networks in the noise variance, components were manually matched following visual inspection. Spatial correlation was used to quantify the similarity of network patterns in the noise variance and the cleaned data, and in the cleaned data relative to network structures provided in the literature (Smith et al., 2009). An alternative metric in the literature (Moodie et al., 2014; Tie et al., 2014; Wisner et al., 2013; Zhu et al., 2013), Dice's coefficient, was also calculated for these network pairs using thresholded maps (thresholded p < 0.05, using the alternative hypothesis testing and mixture model approach within the MELODIC framework). In the simulated noise data and reduced data, the maximum spatial correlation was used to identify matching components in each iteration. The iteration reflecting the median spatial correlation across all iterations was identified, and the network map presented (thresholded Z > 3.0) in the figures.

### Variance analysis

The variance explained (and removed) by true and simulated noise regressors was calculated within the 9 functional networks of interest observed in the cleaned data. Network maps from the cleaned data were thresholded (Z > 3.0) to form region of interest masks. The AFNI software used to perform GLM fitting in the Construction of “noise” datasets section automatically output maps of voxelwise R^2^ values, representing the variance explained by the full noise model. The median voxelwise R^2^ value was calculated within the network-based ROI masks for each combination of true and simulated noise regressors and for each subject, using the first iteration of phase-randomised simulated noise regressors for simplicity. Additional GLM analyses were performed to test combinations of true and simulated noise regressors: 3 true (translations) and 9 simulated, 6 true (translations and rotations) and 6 simulated, 3 true (translations) and 21 simulated, and 6 true (translations and rotations) and 18 simulated head motion regressors.

## Results

### Networks observed in true noise variance

**Fig. 2:**
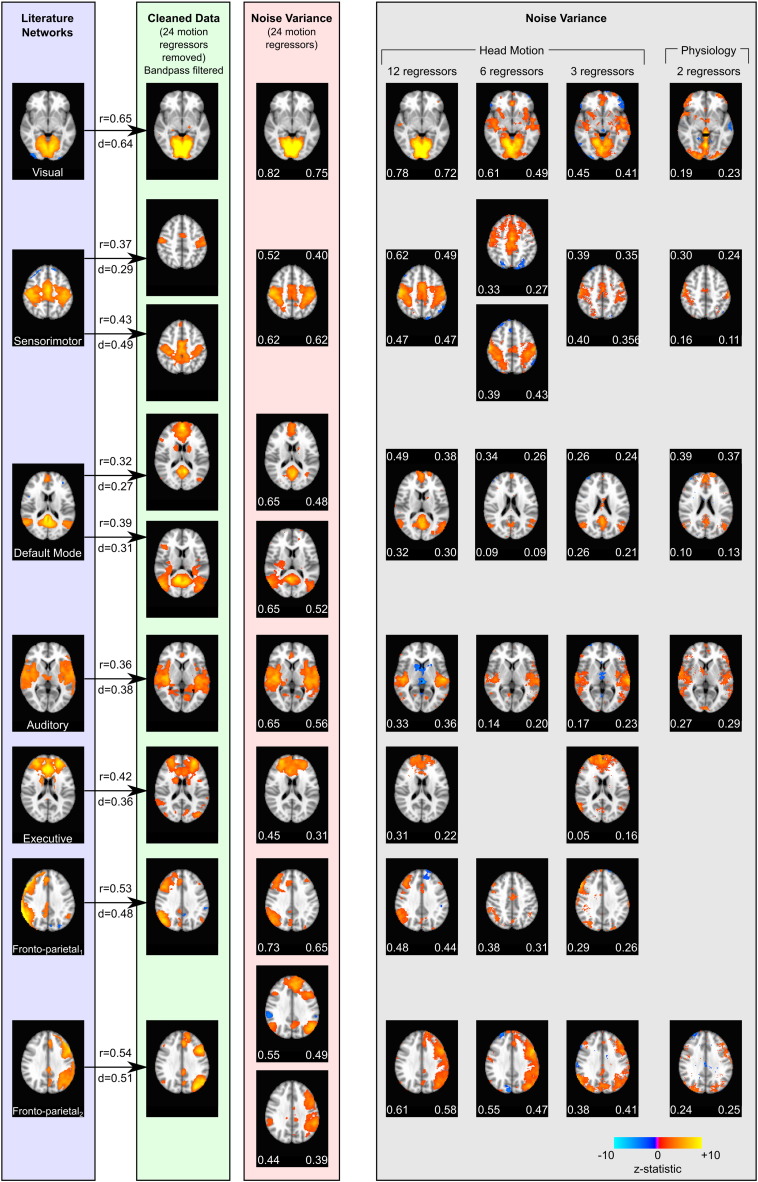
Networks identified in resting state functional connectivity studies (Smith et al., 2009, PNAS 106 (31):13040–5) are observed in the cleaned data (24 head motion nuisance regressors removed). Spatial correlation (r) and Dice's coefficient (d) are provided to quantify similarity between network maps. The 9 networks observed in the cleaned data are also observable in the *noise* variance removed by the 24 motion regressors. Many of these networks continue to be observed in the noise variance as the number of contributing nuisance regressors is reduced (Motion12, Motion6, and Motion3 datasets, as well as variance associated with 2 physiological regressors). Spatial correlation (left values) and Dice's coefficients (right values) quantify the similarity of the noise dataset networks and the cleaned data networks. Some networks are observed to split into two components, merge into one component, or are not present at all within certain noise datasets.

In the data variance extracted by the true noise regressors (typically removed from fMRI data during pre-processing), we observed numerous network structures similar to those identified in the functional connectivity literature and in the cleaned data. Fig. 2 demonstrates the breadth of network structure identified in the variance removed by different combinations of nuisance regressors, including visual, sensorimotor, executive, auditory, fronto-parietal and default mode networks. Spatial correlation values and Dice coefficients show that networks in the noise variance are in good agreement with the networks identified in the cleaned data, which in turn can be matched to established functional networks from the resting state literature (Smith et al., 2009). In the noise variance reflecting 24 motion regressors, spatial correlation values ranged from 0.44 to 0.82 across the 9 networks examined; values above 0.25 are typically considered significant in the literature (Forman et al., 1995; Smith et al., 2009; Taylor et al., 2012). A similar figure using the unfiltered cleaned data as the reference is presented in the supplementary material (Fig. S3).

In the data variance extracted by the true noise regressors (typically removed from fMRI data during pre-processing), we observed numerous network structures similar to those identified in the functional connectivity literature and in the cleaned data. Fig. 2 demonstrates the breadth of network structure identified in the variance removed by different combinations of nuisance regressors, including visual, sensorimotor, executive, auditory, fronto-parietal and default mode networks. Spatial correlation values and Dice coefficients show that networks in the noise variance are in good agreement with the networks identified in the cleaned data, which in turn can be matched to established functional networks from the resting state literature (Smith et al., 2009). In the noise variance reflecting 24 motion regressors, spatial correlation values ranged from 0.44 to 0.82 across the 9 networks examined; values above 0.25 are typically considered significant in the literature (Forman et al., 1995; Smith et al., 2009; Taylor et al., 2012). A similar figure using the unfiltered cleaned data as the reference is presented in the supplementary material (Fig. S3).

As apparent in Fig. 2, network structure continues to be present in the true noise data as the number of contributing nuisance regressors is reduced from 24 to 2, although not every network is discernable in every noise dataset examined and the splitting of the motor, default mode, and fronto-parietal networks varies. Both similarity metrics as well as qualitative agreement reduce with the number of contributing regressors; spatial correlations generally reduce to non-significant levels (r < 0.25) in the case of 2 nuisance regressors. ICA results from the true noise data and cleaned data presented in Figs. 2 and S3 are available as NIFTI files in the supplementary material.

As apparent in Fig. 2, network structure continues to be present in the true noise data as the number of contributing nuisance regressors is reduced from 24 to 2, although not every network is discernable in every noise dataset examined and the splitting of the motor, default mode, and fronto-parietal networks varies. Both similarity metrics as well as qualitative agreement reduce with the number of contributing regressors; spatial correlations generally reduce to non-significant levels (r < 0.25) in the case of 2 nuisance regressors. ICA results from the true noise data and cleaned data presented in Figs. 2 and S3 are available as NIFTI files in the supplementary material.

### True versus simulated noise

**Fig. 3:**
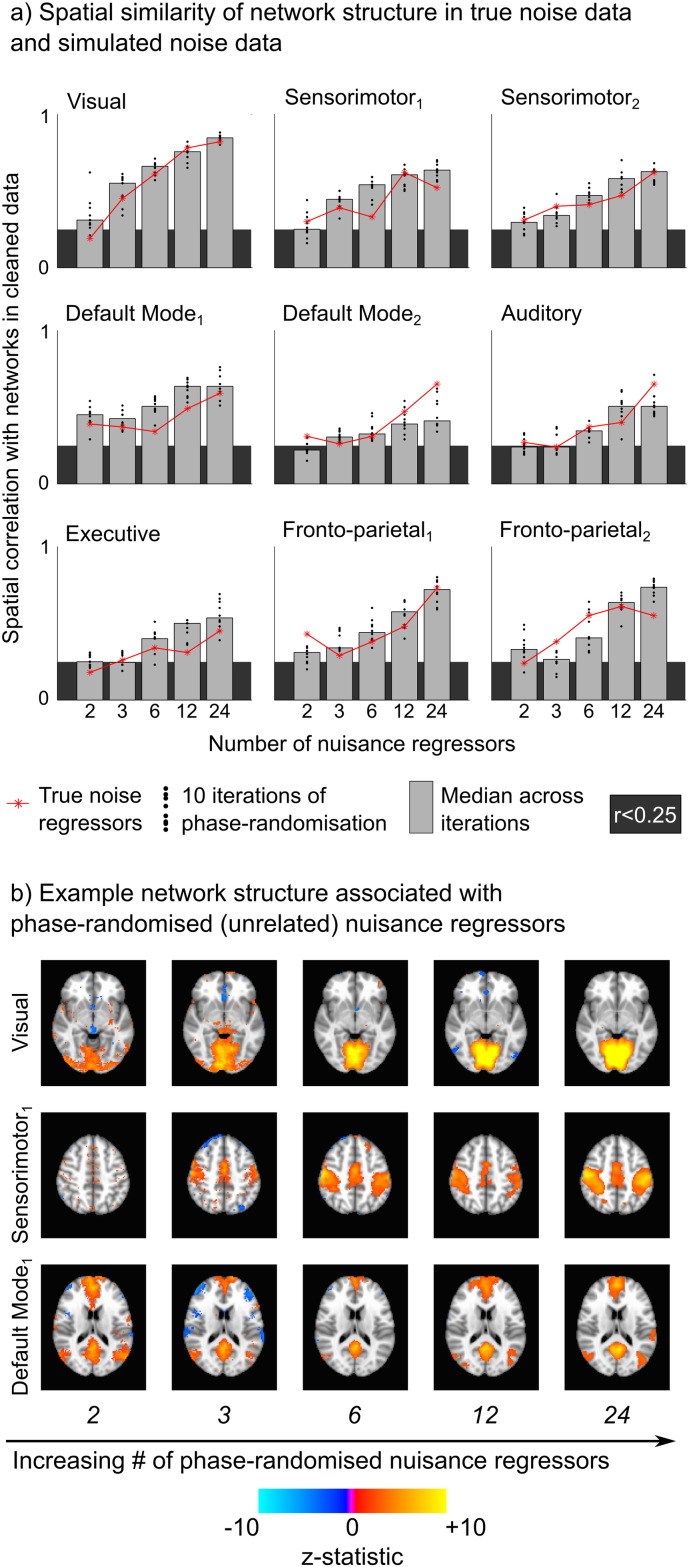
a) Spatial correlation comparing the networks in the cleaned data to those in true noise data (red lines) or simulated noise datasets (black points representing 10 iterations, median value indicated by grey bars). Dark grey shading indicates non-significant similarity (r < 0.25). Both true and simulated noise contain these two networks with high fidelity in the case of 12 or 24 nuisance regressors, suggesting that the network structure in the noise variance is not inherently linked to true noise and can be achieved via unrelated (orthogonalised) nuisance regressors. b) The maps demonstrating the median spatial correlation value across the 10 iterations of simulated noise datasets is provided for 3 networks (Visual, Sensorimotor_1_ and Default Mode_1_).

Maximum spatial correlation with the cleaned data networks was used to identify nine components in the simulated noise datasets (for each of the 10 iterations). Fig. 3 summarises the similarity between the true/simulated data networks and the cleaned data networks. The simulated noise data contain network structures with similar spatial correlation to the cleaned data maps as the true noise data, suggesting that the network structure in the noise variance is not inherently linked to either true head motion or true physiological noise and can be achieved via unrelated (orthogonalised) nuisance regressors.

Due to the way simulated noise regressors were derived (i.e., forced orthogonality), statistical tests for significant differences between these results are not valid. However, for most networks the simulated noise data show a monotonically increasing relationship between the spatial similarity relative to the cleaned data map and the number of nuisance regressors contributing to the simulated noise dataset. This demonstrates the potential impact of the “degrees of freedom” associated with nuisance regressors on the network-related variance extracted by the regression. This concept is revisited in the Network structure in random sampling of fMRI variance section and Variance explained by true and simulated noise regressors section.

Finally, the results from the noise data using true regressors from a different subject's data are presented in Supplementary Fig. S4, providing additional evidence that nuisance regressors unrelated to the data can remove variance of interest, even with minimal numbers of regressors (degrees of freedom) used in the fitting procedure.

Finally, the results from the noise data using true regressors from a different subject's data are presented in Supplementary Fig. S4, providing additional evidence that nuisance regressors unrelated to the data can remove variance of interest, even with minimal numbers of regressors (degrees of freedom) used in the fitting procedure.

### Network structure in random sampling of fMRI variance

**Fig. 4:**
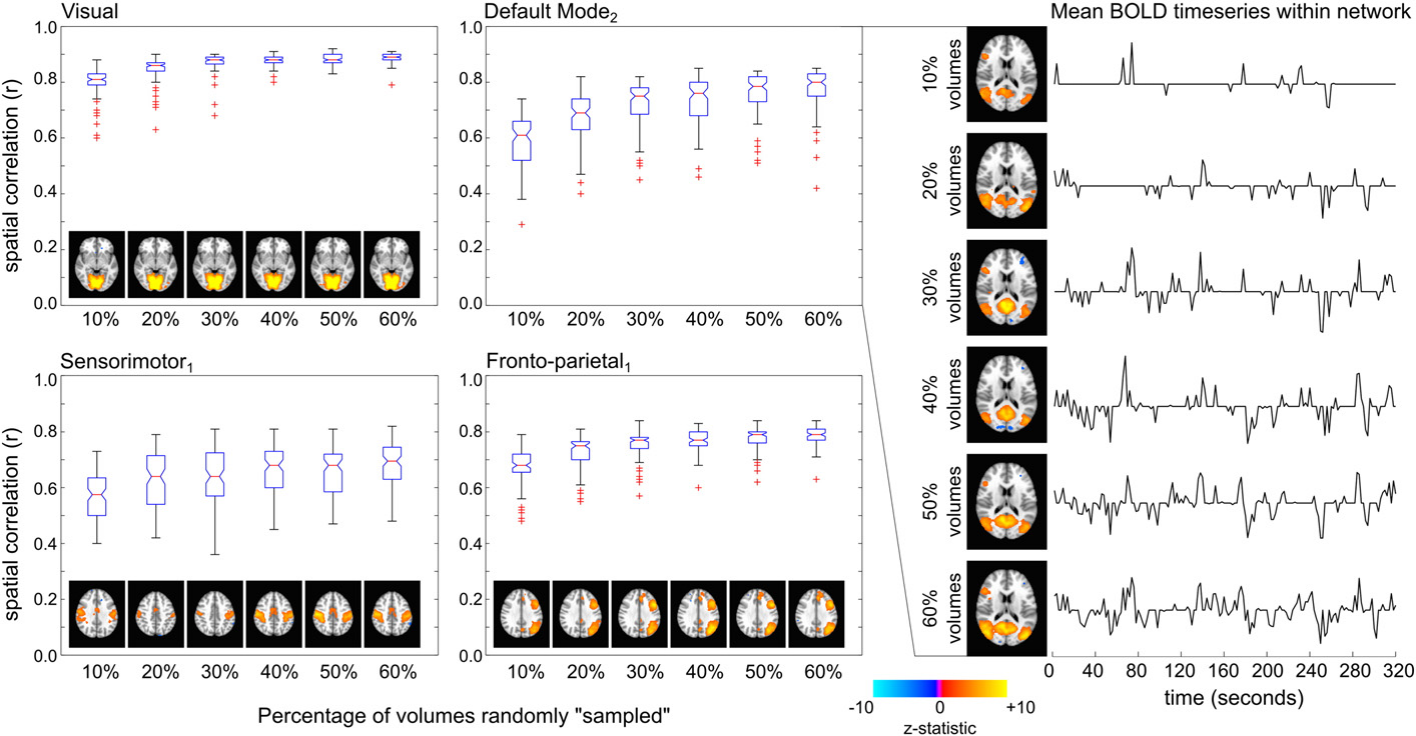
Spatial correlation between the reduced data network maps and the cleaned data maps for 4 of the 9 networks. Boxplots represent the median and quartile distributions of spatial correlation coefficients (r) for the 100 iterations of randomly reduced data. The network maps associated with the median correlation values are provided for reference. The reduced60 and cleaned data demonstrated significant spatial correlation for all 9 networks examined. The mean time-series within the default mode network for one subject is shown for the 6 reduced datasets (map thresholded Z > 3 before signal averaging within this mask).

Because we observe network structure in the variance associated with simulated nuisance regressors, it is likely that random sampling of the fMRI signal variance is sufficient to observe the same phenomenon. Using the reduced data to test this hypothesis, we observed network structure in subsets of the data with as few as 10% of volumes (16 volumes per subject) randomly “sampled.” Fig. 4 shows the spatial correlation between the reduced data network maps and the cleaned data maps for 4 networks. The boxplots represent the median and quartile distributions of spatial correlation coefficients (r) for the 100 iterations of randomly reduced data. The network maps associated with the median correlation values are provided for reference. The mean time-series within the default mode network for one subject is shown for the 6 reduced datasets (map thresholded Z > 3 before signal averaging within this mask). There is little decrease in spatial correlation as the data is reduced from 60% to 10% of the original volumes.

**Fig. 5:**
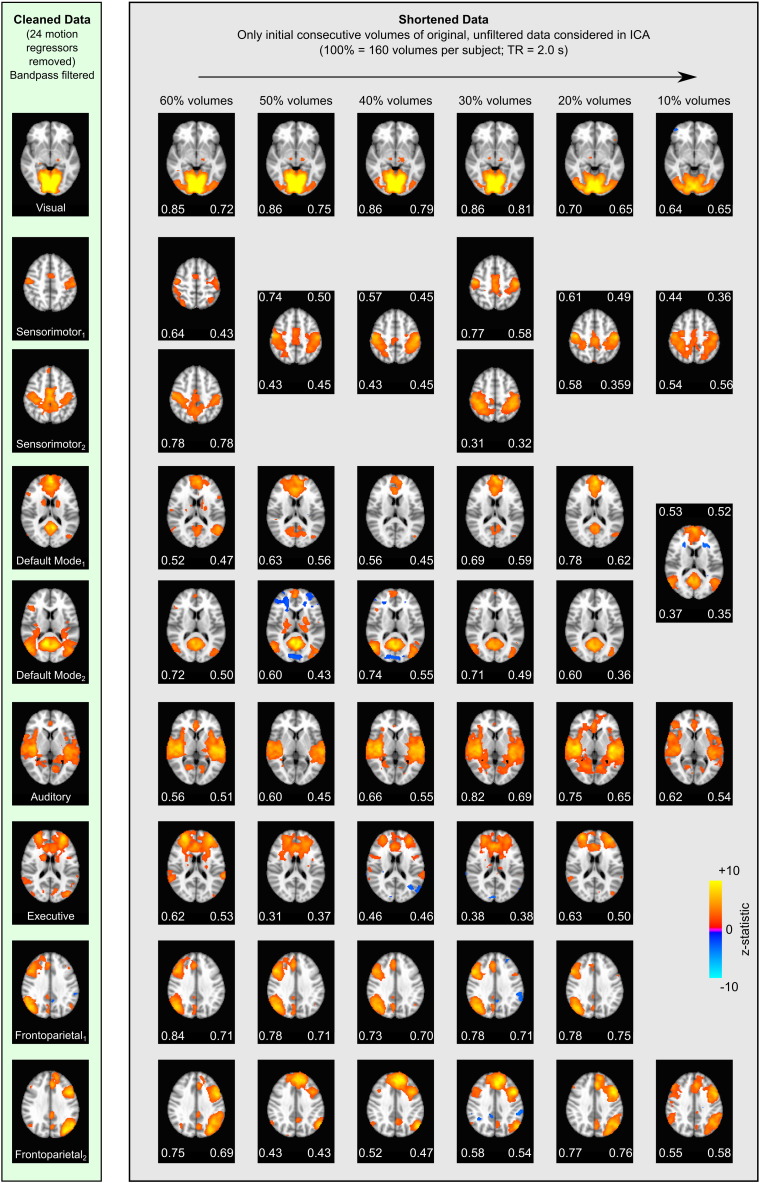
The networks observed in the total cleaned datasets are also observable in the shortened data consisting of only the initial 10–60% of consecutive volumes in each subject's original unfiltered data. Spatial correlation (left values) and Dice's coefficients (right values) quantify the similarity of the shortened dataset networks and the cleaned data networks. Some networks are observed to split into two components, merge into one component, or are not present at all. All networks are identified in the short20 data (32 volumes, 64 s data per subject).

This raises the important question of whether substantially shortened scans could achieve spatial maps of functional networks with fidelity. Fig. 5 demonstrates that the 9 networks observed in the total cleaned dataset are also observed in the shortened data, with as few as the initial 20% of volumes (32 volumes, approximately 1 min of scan time) considered for each subject. The default mode network, visual network, motor network, and auditory network are also visible in the short10 data, which represents 32 s of fMRI acquisition for each subject. The remaining networks are not visible, potentially as a result of the limited dimensionality in the ICA decomposition.

The results of these toy examples indicate that network structure is extremely robust, and can be extracted from small subsets of the original data variance.

### Variance explained by true and simulated noise regressors

**Fig. 6:**
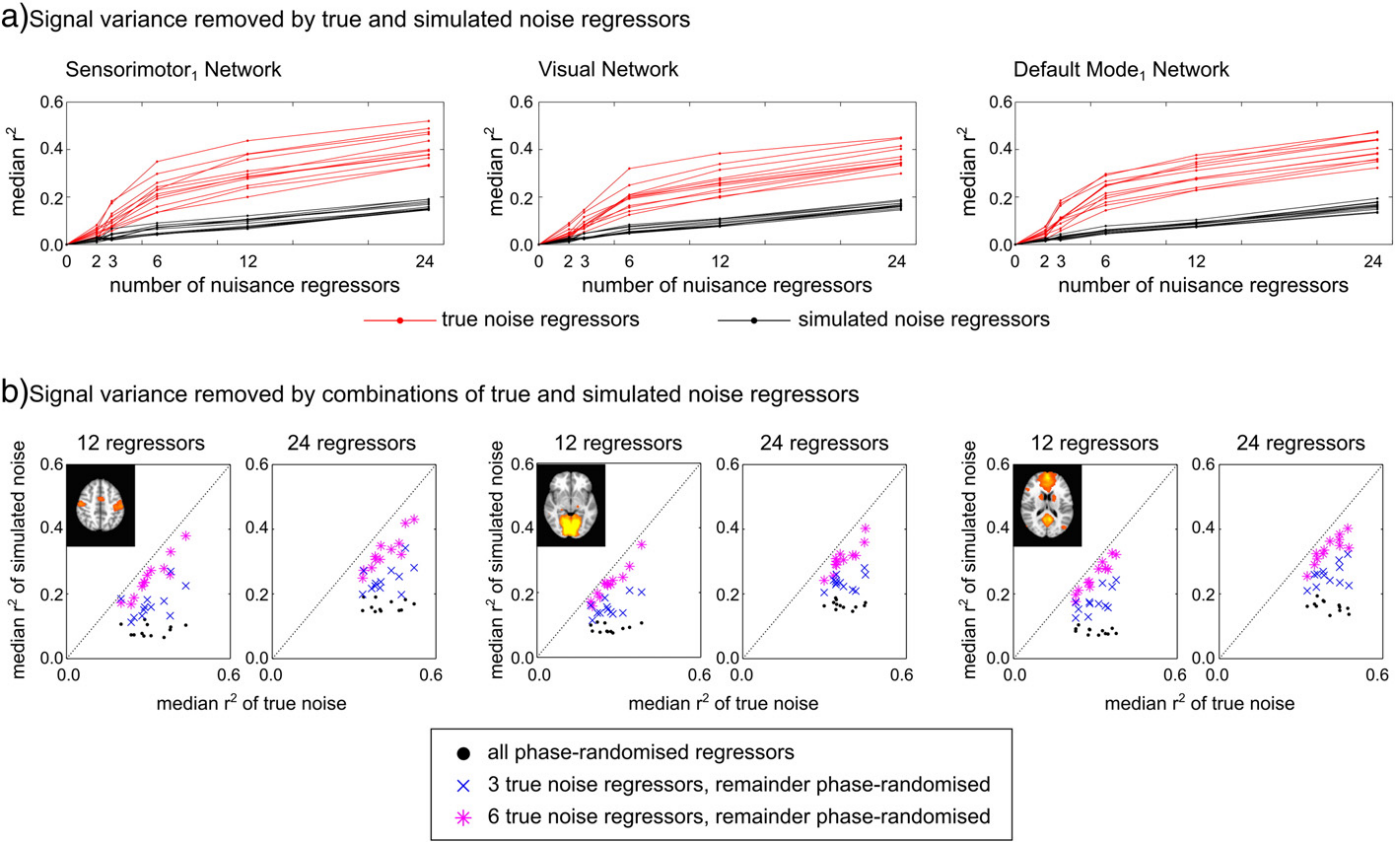
(a) Signal variance removed by true and simulated noise regressors, quantified as the median R^2^ value across voxels within the network mask in the cleaned data. The results for true noise (red lines) and simulated noise (black lines, the 1st iteration only) are presented for each of 12 subjects and within 3 networks (Sensorimotor_1_, Visual, and Default Mode_1_). True noise regressors remove more variance than simulated noise regressors in all cases, and the amount of variance removed increases as the number of nuisance regressors increases. (b) Comparison of the variance explained by the true noise regressors and combinations of true/simulated noise regressors, controlling for degrees of freedom. The correlation across the 12 subjects is non-significant when comparing the variance explained by all true and all simulated regressors. However, when 6 simulated noise regressors are replaced with 6 true noise regressors (translations and rotations), significant correlation is observed in all networks in the case of 12 or 24 total nuisance regressors (p < 0.05, corrected for 18 comparisons; note one non-significant finding was observed in the case of 24 nuisance regressors in the Fronto-parietal_2_ network).

In Fig. 6a we demonstrate that true noise regressors remove consistently more variance from the original BOLD fMRI data than the simulated noise regressors, as may be expected given that true noise regressors reflect theoretical and empirical noise models designed to remove known signal confounds from resting state data. In addition, the amount of variance explained increases as the number of nuisance regressors increases, whether true or simulated, as expected when increasing the degrees of freedom in the GLM fitting procedure.

We also observe a change in the relationship of additional true/simulated noise regressors and the amount of BOLD variance explained when considering few (2–6) compared to many (12–24) nuisance regressors. Specifically, the addition of up to 6 nuisance regressors results in sharp deviation between the amount of variance explained by true versus simulated noise regressors, while beyond 6 regressors there is a more similar increase in the variance explained. This suggests that beyond 6 true noise regressors (translations and rotations), the addition of more true noise regressors to the GLM is akin to adding unrelated simulated regressors with respect to the amount of variance removed within a functional network.

To test this, Fig. 6b presents the correlation between variance explained by combinations of true and simulated noise regressors compared with the variance explained by all true noise regressors, while maintaining the total number of nuisance regressors (12 or 24) to control for degrees of freedom in fitting. There is no correlation across subjects in any network examined between the variance explained by all true and all simulated noise regressors (black dots). Replacing 3 simulated regressors with the true 3 head motion regressors (x-, y-, and z-translations) does not improve this. However, replacing 6 simulated regressors with the true 6 head motion regressors results in significant correlation (p < 0.05, corrected for 18 comparisons) with the variance explained by all true noise regressors. (Note, one non-significant finding was observed in the case of 24 nuisance regressors in the Fronto-parietal_2_ network). These results quantitatively support the qualitative observation in Fig. 6a: in our data, beyond 6 true head motion regressors, additional regressors may act similarly to unrelated simulated nuisance regressors, which function to randomly sample (and remove) variance.

## Discussion

### Are functional networks inherently associated with true noise?

Given that we observe network structure in the true noise datasets (Fig. 2), it is important to consider whether there may be a real relationship between the neural activity in these functional networks and the noise sources modelled by our nuisance regressors.

In this study, the network structure is most robust in the noise variance associated with 12 or 24 head motion regressors. Yan and colleagues (Yan et al., 2013a,b) proposed that the relationship between motion regressors and the BOLD signal partly reflect a neural signature of head motion rather than strictly artefact. In support of this, recent studies have shown that head motion is linked to signal changes in certain brain regions, including sensory, motor, visual and default mode network areas (Pujol et al., 2014), or may be associated with an inherent subject-specific trait linking connectivity and tendency for head motion (Zeng et al., 2014).

Regarding physiological noise regressors, voluntary breathing has been linked to activation of bilateral sensory and motor cortices (McKay et al., 2003). Cerebrovascular reactivity to arterial CO_2_ changes, independent of neural activation, may also result in BOLD signal changes coupled to our physiologic regressors. There are consistent regional differences in the vascular response to this CO_2_, with areas such as sensorimotor and visual cortices distinguished by early or delayed response times (Bright et al., 2009).

Although these factors do allow for the possibility of an inherent link between true noise and network structure, it seems unlikely that this is the driving source of our observations. Simulated noise regressors that are orthogonalised (and thus unrelated) to the true noise regressors result in similar network decomposition, and often these networks are as similar or more similar to the cleaned data networks than are the true noise equivalents (Fig. 3a). This suggests that any set of nuisance regressors may extract variance that can be decomposed into network structure.

The reduced data analysis (Fig. 4) supports this finding, demonstrating that even very small (10%) sampling of the original data, which is akin to randomly sampling data variance with unrelated nuisance regressors (as in Fig. 6a), will result in similar network structure as the cleaned data. The literature demonstrates that true noise regressors remove signal variance associated specifically with the intended noise confound (see, for example, (Power et al., 2014)); however, we assert that these regressors will also inherently remove some additional variance at random, and that this random sampling of the data drives the observation of interesting network structure in the associated noise datasets. We also assert that this randomly sampled variance must be considered “signal” because it contains multiple, distinct “intrinsic connectivity network” structures: our model of nuisance regressors fits different variance in, say, the Default Mode Network compared to the Visual Network compared to the Sensorimotor Network. Under the assumption that intrinsic connectivity networks are neural networks that express synchronised neural activity, rather than areas where motion confounds are expressed differently due to differences in gyri structure and other brain boundaries (for example), we must also conclude that the variance we are extracting to differentiate these networks represents neural signals as well.

Finally, we observe a wide range of network structures, including short and long-distance nodes across many cortical areas, both bilateral and lateralised, extending beyond the regions indicated in the recent literature as having potential for neural linkage between motion “noise” models and neural activity (Pujol et al., 2014; Zeng et al., 2014).

The complete picture is potentially complicated: the relationship between intrinsic connectivity networks and true noise sources is likely to be variable across different brain regions. However, we observe network structure in nearly all cases examined in this paper. It may be important to characterise the variance that is removed from resting state analysis by nuisance regressors, to assess the impact of how many and what type are used, and to determine whether this is problematic for a given subject or cohort.

### Robustness of intrinsic connectivity networks

In the cleaned data and the true and simulated noise data, we consistently identified network structure (Figs. 2 and 3). In the true noise variance, 12–24 head motion regressors removed variance that exhibited all functional network structure identified in the cleaned data. Considering fewer nuisance regressors (or fewer volumes, as in the short10 data of Fig. 5), the ICA decomposition identified some, although not all, network structures. This potentially reflects sub-optimal decomposition due to fixed ICA dimensionality in situations with differing degrees of freedom in the data, or closer coupling between those true noise regressors and true signal confounds that are spatially unrelated to network structures.

Fig. 4 demonstrates that random sampling of only 16 volumes per subject was sufficient to identify most of these same functional networks in our data. Randomly reducing the data to 10% of volumes is similar to sampling 10% of the total data variance, demonstrating that fMRI “signal” with interesting network structure is likely fit and isolated even when nuisance regressors are removing only very small amounts of variance at random. We also observe that 2 true physiologic or 3 true head motion regressors sample slightly less than 10% of the variance in the data of most subjects and networks examined in this study (Fig. 6a), representing both true noise variance as well as randomly extracted variance, and not all functional brain networks are observable in these true noise datasets. Combined, these results indicate that network structure is sometimes observable in less than 10% of our data variance, but only becomes robust and consistent when 10% or greater random variance is considered.

This suggests that the structure of functional networks—the identification of nodes that exhibit temporally correlated BOLD signal fluctuations in the resting state—is extremely robust beyond this threshold of 10% of our data (16 volumes, 32 s, per subject). By contrast, in the shortened data that considers only the initial consecutive volumes, we observe that 20% of volumes (32 volumes, 64 s, per subject) are necessary to discern all networks considered in this study. The “shortened” data analysis examines how little *data* is needed to observe networks, whereas the “reduced” data analysis examines how little *variance* is needed to observe these networks in a full length dataset. Using consecutive volumes to differentiate intrinsic signal fluctuations may also be less efficient at extracting robust network structures than using randomly sampled volumes, particularly if these fluctuations are at low frequencies of approximately 0.01 Hz. Noise confounds, truly unrelated to the intrinsic signal fluctuations of interest, are also likely to be of more concern in consecutive volumes, potentially hindering our ability to discern the network structures. We conclude that ~ 1 min of the initial data is necessary to observe robust network structure in this study, although randomly sampling from a longer dataset may require less data.

This initially appears at odds with the literature, where it has been suggested that between 5 and 12 min of data (Birn et al., 2013; Van Dijk et al., 2010), or 3 min of “scrubbed” data (Yan et al., 2013a), is desirable for reliable connectivity estimates. These previous studies, however, have assessed connectivity measures such as temporal correlation, whereas here we examine spatial structure. Note also that we include data from 12 subjects in the ICA decomposition, and thus approximately 12 min of data are used in the automatic extraction of network structure in this paper, despite needing very little data from an individual subject.

Within the resting state literature, temporal correlations are commonly used to map network structure, and correlation strengths are then calculated within this structure—a potentially circular procedure. Knowing that a small number of fMRI volumes (~ 1 min with a TR of 2 s) are sufficient to map network structure in a population, we could use short scans to spatially define networks independently of the temporal correlations in a longer functional scan and prevent the possibility of “double dipping” in our analysis. Alternatively, a random sampling of an even smaller number of volumes could be extracted from a longer resting-state dataset for this purpose. Both approaches could be applied to existing data with minimal cost.

### Implications for nuisance regressors

In this study, we highlight the potentially detrimental aspects of using nuisance regressors to remove noise from resting-state data; particularly, that increasing the number of nuisance regressors is more likely to result in the removal of variance containing “interesting” structure. Here we examine the variance removed by the different noise models in the context of the field, and how nuisance regressors could be limited to reduce over-fitting of interesting signal variance.

#### Variance explained by true noise regressors

In the original paper proposing the cardiac rate noise correction technique, multiple lagged heart rate regressors were observed to explain 1.5% of variance beyond that explained by polynomial and RETROICOR regressors (Shmueli et al., 2007); this underestimates the contribution of heart rate in the current study, where we consider only 1st and 2nd order polynomial terms and do not consider RETROICOR. In the original paper proposing end-tidal CO_2_ as a nuisance regressor, it was observed to explain 4.7% of variance (Wise et al., 2004) within grey matter, although this study only considered voxels significantly correlated to the end-tidal CO_2_ regressor and thus overestimates this effect. In our study, we observe that combined, these physiological regressors, in conjunction with linear and quadratic detrending terms, explain approximately 5–10% of signal variance across subjects, which is in agreement with the literature reports albeit difficult to make a fair comparison. We observe that 6 motion regressors explains slightly more variance (approximately 10–30%) than reported elsewhere (~ 5–20%, (Jo et al., 2010)), however, differences in calculating the mean/median across voxels, using voxelwise values or considering mean time-series, examining all grey matter versus smaller regions of interest, and the use of bandpass filtering may affect specific quantification. To clarify for future comparisons, the values presented here represent the median voxelwise R^2^ value for the entire nuisance regressor model fit, including linear and quadratic detrending terms, across network regions of interest defined elsewhere, with no additional grey/white-matter masking, and no bandpass filtering.

#### How many nuisance regressors to use?

The concern over the expanding number of nuisance regressors in resting state fMRI pre-processing and the impact on degrees of freedom has been raised in the literature. Satterthwaite and colleagues tempered their proposed 24-regressor model with the caveat that connectivity values do not yet take into account the degrees of freedom contributing to that value (Satterthwaite et al., 2013). Others have applied Principal Component Analysis to reduce 6 motion regressors to 2, still containing up to 85% of the original motion-related variance but with a smaller impact on degrees of freedom (Churchill et al., 2012). Similarly, Jo and colleagues state that the number of physiological nuisance regressors (e.g., RETROICOR (Glover et al., 2000)) should be reduced because they do not explain much variance compared to their effect on degrees of freedom (Jo et al., 2010).

Precisely where to draw the line between beneficial and confounding nuisance regression is inherently ambiguous. In our data, we find that more than 6 motion regressors remove a similar amount of variance to unrelated simulated noise regressors. If we make the assumption that simulated or random regressors remove variance at random while true noise regressors remove both the intended noise variance as well as some degree of random variance, the fact that the amount of variance removed is similar in these two scenarios demonstrates the inherent difficulty in quantitatively proving that additional true noise regressors perform “better” than random equivalents. In addition, the variance explained by just 6 or fewer regressors did not demonstrate as robust network structure as 12 or 24 regressors (Fig. 2), indicating that analysis of our data may be best served by limiting the number of motion-related nuisance regressors to 6.

Besides reducing the size of the nuisance regressor model, there is an alternative means of addressing the balance between beneficial de-noising and random removal of signal. As the underlying concern is one of degrees of freedom, the problems associated with excessive regression can be attenuated by extending the number of time-points in the data. This study considers data with 160 time-points, and observes that approximately 6 motion regressors may be optimal; however, longer datasets with many hundreds of fMRI volumes are likely to be well-suited for increased numbers of nuisance regressors.

**Fig. 7:**
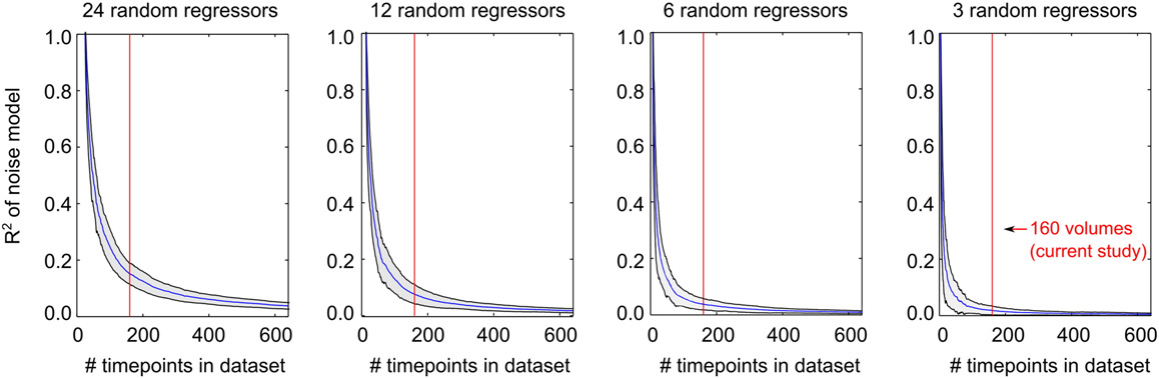
Simulation of the effect of scan length on random sampling of fMRI signal by nuisance regressors. BOLD “signal” was modelled as a low frequency (0.01 Hz) sinusoid, and random nuisance regressors were generated using evenly distributed random numbers. The variance explained by 3, 6, 12, or 24 nuisance regressors (R^2^) is presented, demonstrating how increasing the number of time-points in the fMRI data attenuate the problematic random sampling of signal by nuisance regressors. For reference, the red line indicates 160 time-points, which is the duration of datasets examined in this study.

To test this, we simulated fMRI “signal” as a low frequency sinusoid (0.01 Hz), and examined the variance removed by random nuisance regressors generated using evenly distributed random numbers. The results of 100 iterations (mean and standard deviation) are presented in Fig. 7, demonstrating how the random sampling of signal variance is attenuated as the duration of the fMRI dataset is extended (up to 640 volumes, or 4 times longer than data considered in this study). We observe that the attenuation exhibits a transition or "bend" before asymptotically approaching negligible R^2^ values. At 160 time-points, the variance randomly sampled by 3 or 6 regressors is small and we are operating beyond the "bend" in these plots, whereas 12 or 24 regressors place us within this transition region and the variance being sampled is much higher (approximately 10–20%). Extending the data to 600 or more volumes would ameliorate this concern, bringing us into the asymptotic regime in all plots in Fig. 7. However, note that we have used a very simplistic simulation in which regressors are truly unrelated to the data; in practise, regressor accuracy and total noise levels are likely to influence these attenuation plots.

Together, these findings suggest that limited amounts of physiologic or motion regressors may be the best way forward. We recommend that simulations, similar to those presented here, are applied to determine the most appropriate number of nuisance regressors for a given analysis. For example, regression could be performed on subsets of data with increasing numbers of time-points, to determine if or when the "plateau" is reached. This approach would assist future studies aiming to maximise removal of noise confounds while preserving the existing signals of interest in the data.

We also recommend that future studies characterise the variance removed by any pre-processing nuisance regression, to determine whether this balance is altered in different datasets. For example, some cohorts may display greater head motion artefacts than other cohorts (Van Dijk et al., 2012); in subjects with large amounts of head motion, the addition of extra motion-related nuisance regressors may make the cleaned data more accurate, while in subjects with much less head motion these additional regressors may serve more to randomly sample and remove signals within functional networks. Thus nuisance regressors could create false positives in addition to false negatives in resting state studies.

#### Ambiguity in quantifying network characteristics

The main aim of this paper is to demonstrate that nuisance regressors extract and discard signal variance with network structure. Here we also assess the impact of nuisance regression on temporal correlations between network nodes, using the Default Mode Network as an example. The four largest clusters were extracted from a map of the Default Mode Network taken from the literature (Smith et al., 2009), and the mean %BOLD time-series within these four nodes were extracted in each subject's original, true noise, and cleaned (unfiltered) data. Correlation between each node pair was calculated and converted using the Fisher r–z transform before averaging across subjects. We have presented the resulting group average correlation matrices in supplementary material (Fig. S5), for the Motion24, Motion12, Motion6, and Motion3 analyses.

The main aim of this paper is to demonstrate that nuisance regressors extract and discard signal variance with network structure. Here we also assess the impact of nuisance regression on temporal correlations between network nodes, using the Default Mode Network as an example. The four largest clusters were extracted from a map of the Default Mode Network taken from the literature (Smith et al., 2009), and the mean %BOLD time-series within these four nodes were extracted in each subject's original, true noise, and cleaned (unfiltered) data. Correlation between each node pair was calculated and converted using the Fisher r–z transform before averaging across subjects. We have presented the resulting group average correlation matrices in supplementary material (Fig. S5), for the Motion24, Motion12, Motion6, and Motion3 analyses.

We observe no significant changes in the node-pair correlation values between the original data and the cleaned data using 24, 12, 6, or 3 motion regressors to de-noise, and only one instance of significant difference in the correlations in the cleaned data relative to the corresponding noise data. The lack of significant changes in the temporal correlation values following nuisance regression may mean that, to many users, our observations of network structure in the noise variance are not particularly concerning. However, temporal correlation values are similar no matter what portion of the variance is examined. Given that we can divide our data variance in multiple ways depending on how many nuisance regressors are used, attributing one portion to signal and the other to noise, the consistency of temporal correlations in the “signal” and “noise” variance suggests it is ambiguous to use temporal correlations alone as a marker for optimising pre-processing methods.

#### Future work

Finally, we have provided a new framework for examining the benefits of nuisance regressors by comparing (across subjects) the amount of variance removed by the proposed nuisance regressor relative to the variance removed by a phase-randomised regressor. Simulations could also be used to quantify the amount of data variance expected to be removed purely at random with each additional nuisance regressor added to the noise model, as described in Fig. 7. This approach may contribute towards a test for determining whether a new nuisance regressor removes noise rather than randomly sampled variance, particularly when the intent is to compare the results across subjects with varying noise contributions.

## Conclusion

We have shown that the data variance typically removed and discarded during noise correction of resting state data can be decomposed into network structure highly correlated to networks documented in the functional connectivity literature.

Simulated regressors, unrelated to the true noise, also remove data with network structure. This suggests that any set of regressors may remove highly structured “signal” as well as “noise” and that this may become increasingly apparent as the number of regressors increases.

We have shown that reduced subsets of the original resting state data can also be decomposed into robust network maps using ICA. With as few as 20% of the original volumes considered, we observe spatial network maps similar to those identified in the entire cleaned dataset. These results show that the spatial distribution of intrinsic connectivity networks can be robustly mapped using very sparse temporal sampling of fMRI data.

Finally, we have demonstrated that using excessive true noise regressors related to head motion may be detrimental, removing amounts of variance similar to that of simulated noise regressors while affecting degrees of freedom such that interesting “signal” is removed from the resting state data. This balance between de-noising and signal loss can be adjusted by considering datasets with greater numbers of time-points.

It remains an open challenge to identify when nuisance regressors are no longer beneficial and become an added confound themselves in resting state fMRI.

The following are the supplementary data related to this article.

Fig. S1 True and simulated noise regressors for an example subject, demonstrating the results of the phase-randomisation technique.

Fig. S2 Correlation across the regressors considered in the true noise, simulated noise, and “incorrect subject” noise analyses in this manuscript. For simplicity, only the first iteration of the phase-randomised simulated regressors is included in this figure. For subject N, the true noise regressors from subject N + 1 are used as the “incorrect subject” noise data. Correlation coefficients were transformed using the Fisher r-to-z transform prior to averaging across subjects, and the inverse transformation of the group mean is presented. As intended, minimal correlation structure is observed between the true and simulated noise regressors of a given subject. By design, the true noise regressors and the “incorrect subject” regressors represent the same data and demonstrate identical internal correlation structure; there is also consistent correlation in the regressors across the pairs of subjects considered.

Fig. S3 Networks identified in resting state functional connectivity studies (Smith et al., 2009, PNAS 106 (31):13040–5) are observed in the unfiltered cleaned data (24 head motion nuisance regressors removed). Spatial correlation values (r, left) and Dice's overlap coefficients (d, right) quantify the similarity between the unfiltered cleaned data network maps and networks identified in true noise data.

Fig. S4 Examples of network structure in the noise data created using nuisance regressors from a different (incorrect) subject, and thus unrelated to the BOLD data. Spatial correlation values (r, left) and Dice's overlap coefficient (d, right) quantify the similarity of the networks observed with those present in the correctly cleaned data.

Fig. S5 Correlation matrices representing the temporal relationships between the four nodes of Default Mode Network. Each subject's data were transformed into %BOLD units, and the mean time-series within each of the four nodes was extracted. Correlation was calculated between each node pair, and values were transformed using the Fisher r–z transform prior to averaging across subjects. The inverse transform was performed and results are provided. No significant changes were observed between the correlation values of the cleaned and original data for any number of regressors considered (3, 6, 12, or 24 motion regressors). Only one significant difference was observed between the cleaned and true noise data correlations after correcting for multiple tests (p < 0.05, indicated by asterisk).

Supplementary Data. ICA results for all true noise datasets (2phys, 3motion, 6motion, 12motion, and 24motion) and cleaned datasets (24 motion regressors removed, the resulting data either unfiltered or band-pass filtered to include only frequencies between 0.02 and 0.1 Hz). The data from 12 subjects were normalised to the MNI template space, temporally concatenated and decomposed into 20 spatially independent components using MELODIC (FSL). All files are in NIFTI format and contain the resulting spatial maps for each component.

Supplementary data to this article can be found online at http://dx.doi.org/10.1016/j.neuroimage.2015.03.070.
