# Supplementary figures and images for: Is fMRI “noise” really noise? Resting state nuisance regressors remove variance with network structure
Source: Neuroimage. 2015 Jul 1;114:158–69. doi: 10.1016/j.neuroimage.2015.03.070 (PMC4461310; doi:10.1016/j.neuroimage.2015.03.070)

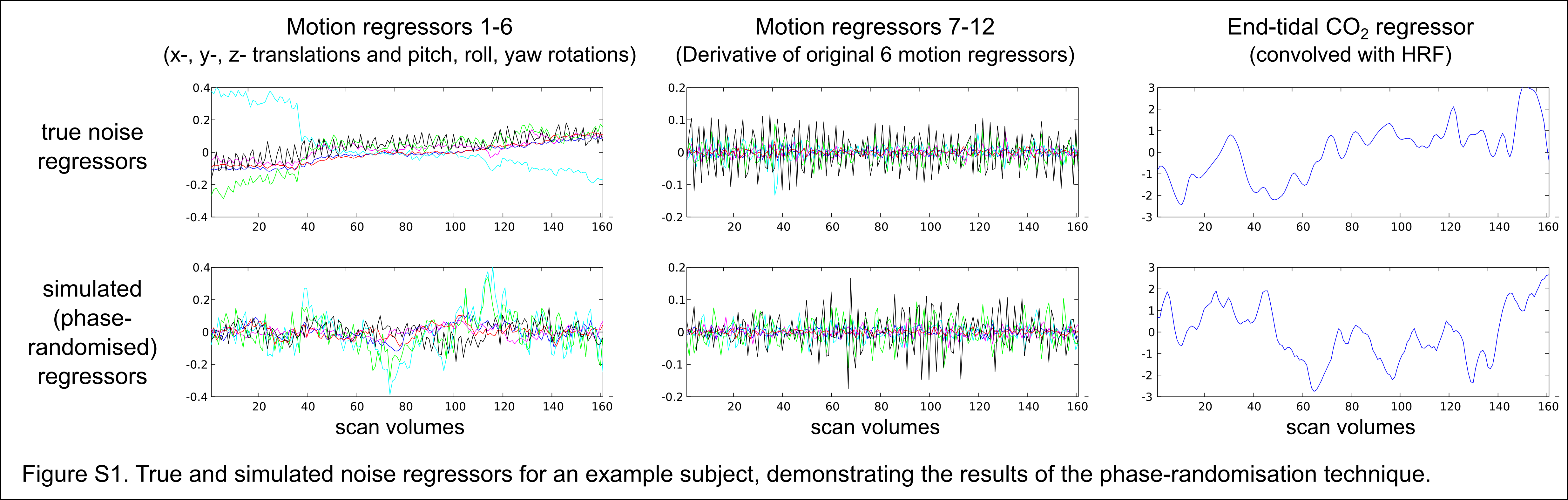

Supplement: Fig. S1 — True and simulated noise regressors for an example subject, demonstrating the results of the phase-randomisation technique. [file mmc1.zip › nim12112-mmc1.png]

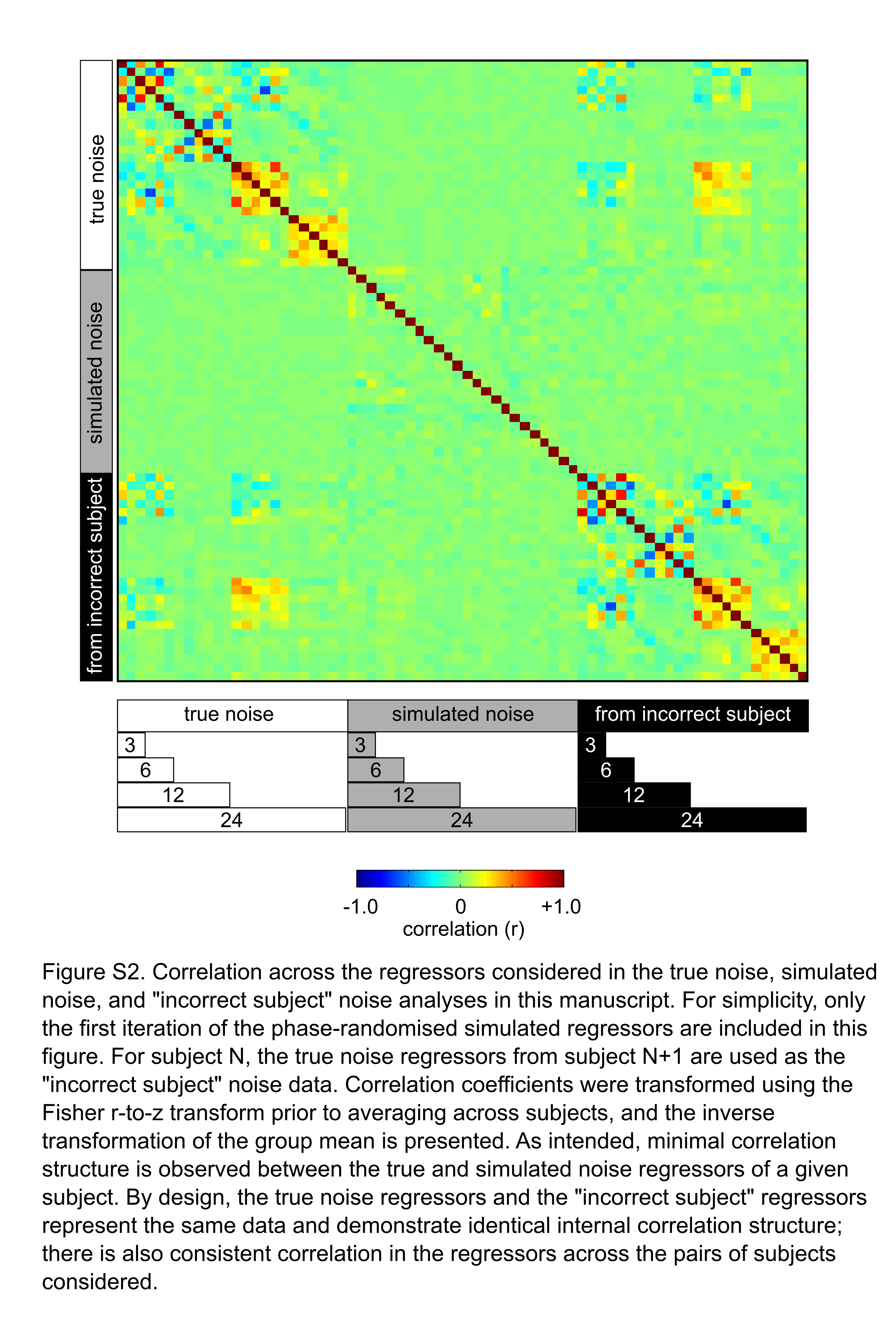

Supplement: Fig. S2 — Correlation across the regressors considered in the true noise, simulated noise, and “incorrect subject” noise analyses in this manuscript. For simplicity, only the first iteration of the phase-randomised simulated regressors is included in this figure. For subject N, the true noise regressors from subject N + 1 are used as the “incorrect subject” noise data. Correlation coefficients were transformed using the Fisher r-to-z transform prior to averaging across subjects, and the inverse transformation of the group mean is presented. As intended, minimal correlation structure is observed between the true and simulated noise regressors of a given subject. By design, the true noise regressors and the “incorrect subject” regressors represent the same data and demonstrate identical internal correlation structure; there is also consistent correlation in the regressors across the pairs of subjects considered. [file mmc2.zip › nim12112-mmc2.png]

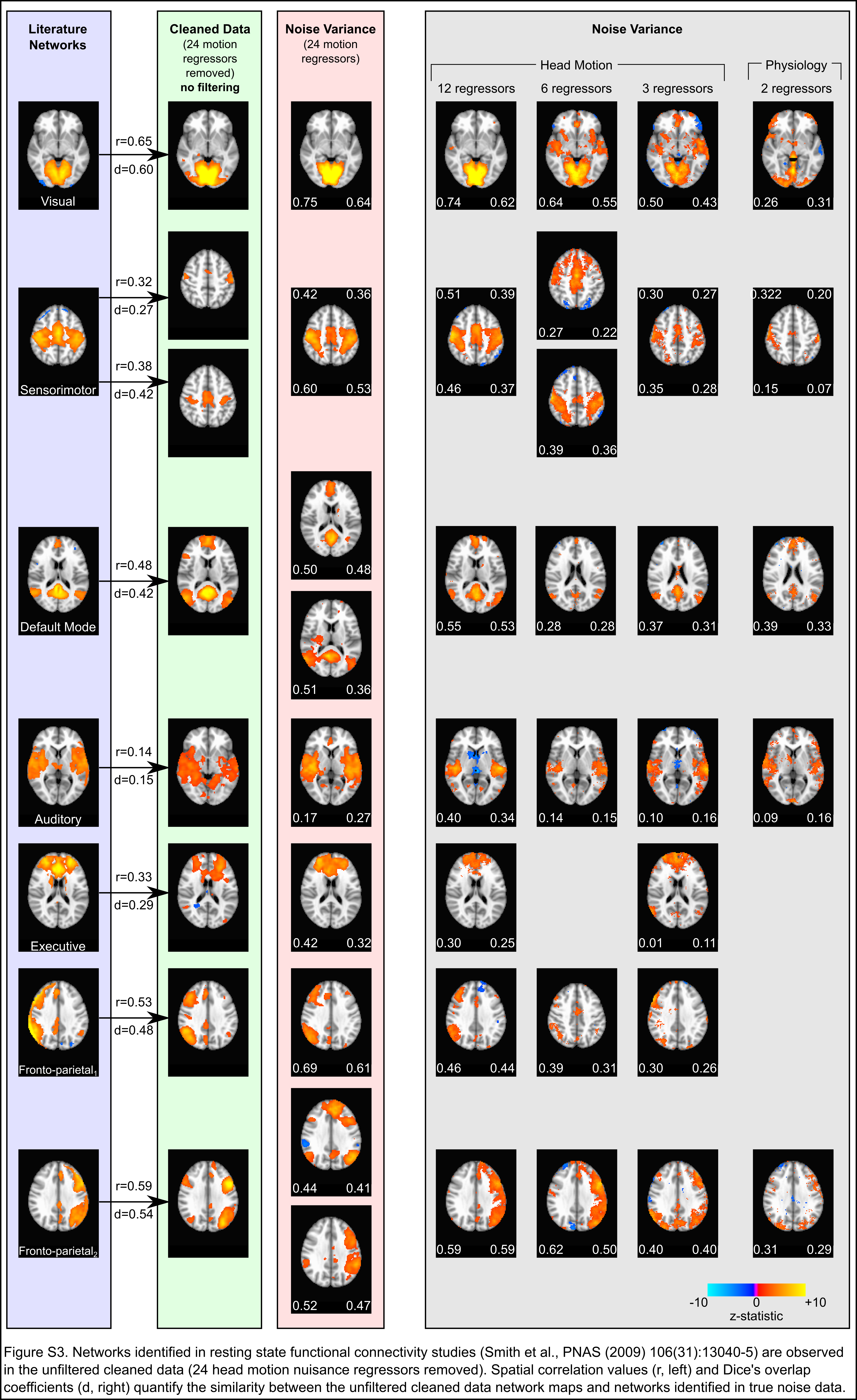

Supplement: Fig. S3 — Networks identified in resting state functional connectivity studies (Smith et al., 2009, PNAS 106 (31):13040–5) are observed in the unfiltered cleaned data (24 head motion nuisance regressors removed). Spatial correlation values (r, left) and Dice's overlap coefficients (d, right) quantify the similarity between the unfiltered cleaned data network maps and networks identified in true noise data. [file mmc3.zip › nim12112-mmc3.png]

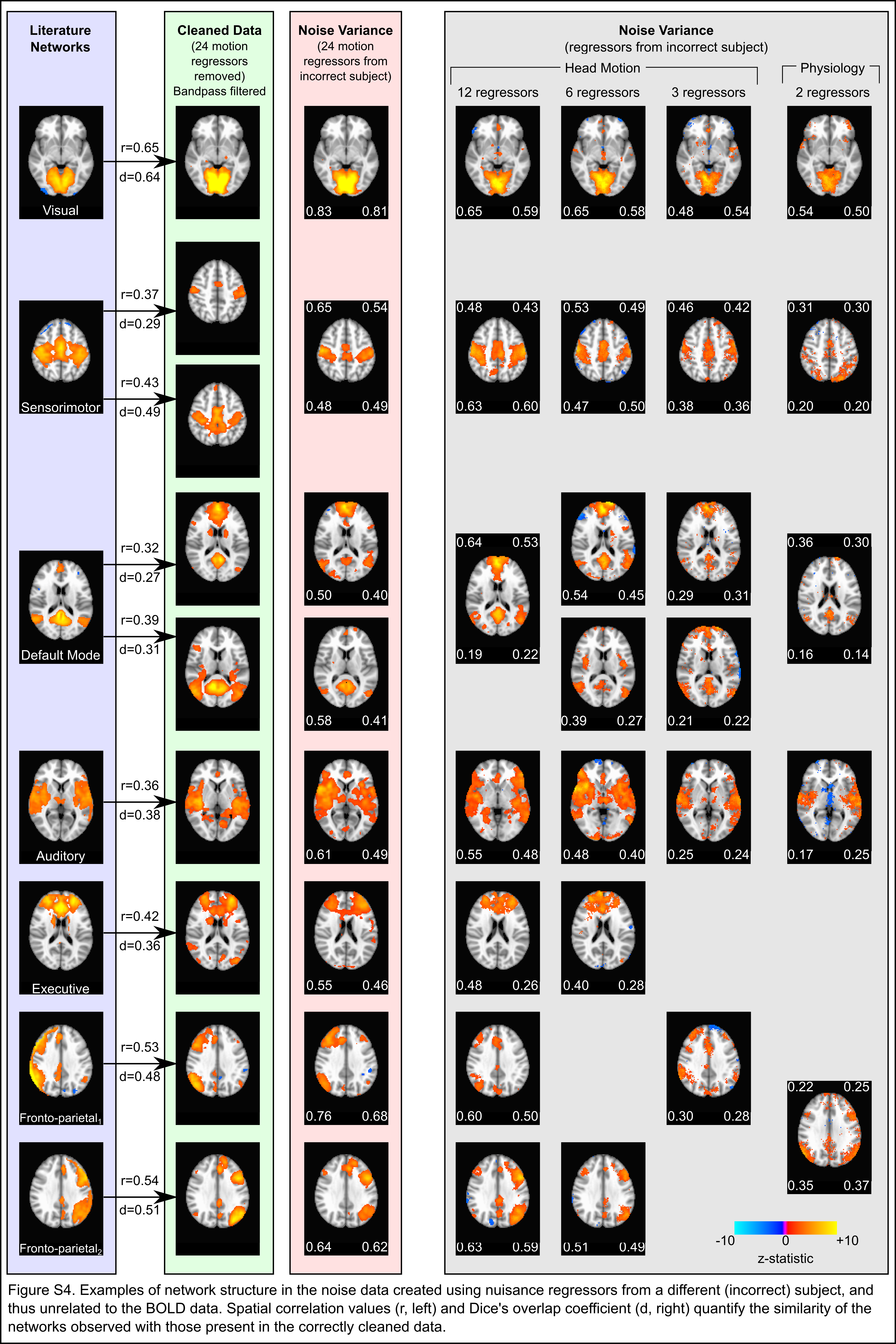

Supplement: Fig. S4 — Examples of network structure in the noise data created using nuisance regressors from a different (incorrect) subject, and thus unrelated to the BOLD data. Spatial correlation values (r, left) and Dice's overlap coefficient (d, right) quantify the similarity of the networks observed with those present in the correctly cleaned data. [file mmc4.zip › nim12112-mmc4.png]

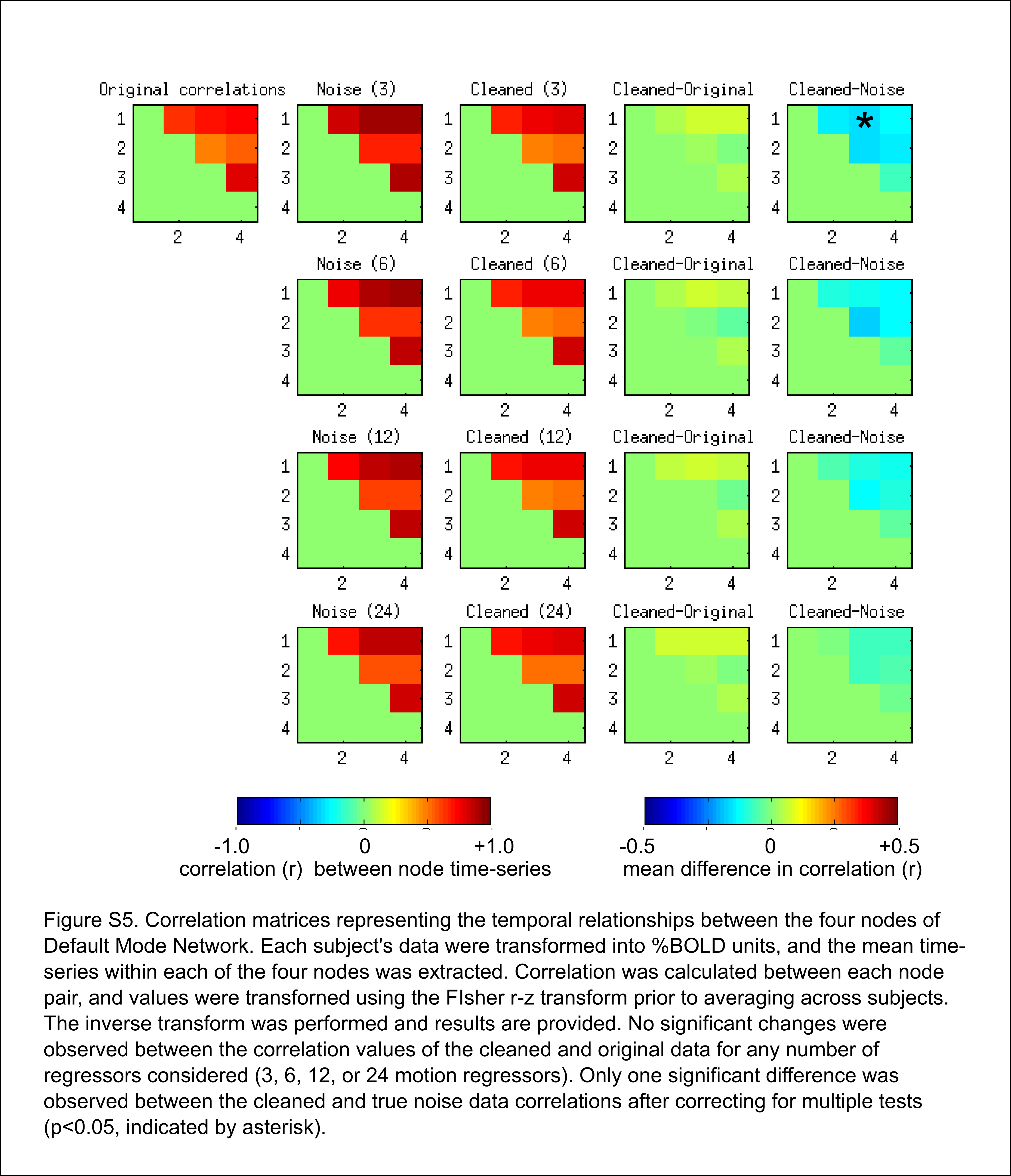

Supplement: Fig. S5 — Correlation matrices representing the temporal relationships between the four nodes of Default Mode Network. Each subject's data were transformed into %BOLD units, and the mean time-series within each of the four nodes was extracted. Correlation was calculated between each node pair, and values were transformed using the Fisher r–z transform prior to averaging across subjects. The inverse transform was performed and results are provided. No significant changes were observed between the correlation values of the cleaned and original data for any number of regressors considered (3, 6, 12, or 24 motion regressors). Only one significant difference was observed between the cleaned and true noise data correlations after correcting for multiple tests (p < 0.05, indicated by asterisk). [file mmc5.zip › nim12112-mmc5.png]
